# Genetic associations between autoimmune diseases and the risks of severe sepsis and 28-day mortality: a two-sample Mendelian randomization study

**DOI:** 10.3389/fmed.2024.1331950

**Published:** 2024-01-26

**Authors:** Xin Tie, Yanjie Zhao, Jing Su, Xing Liu, Tongjuan Zou, Wanhong Yin

**Affiliations:** ^1^Department of Critical Care Medicine, West China Hospital, Sichuan University, Chengdu, China; ^2^Department of Neurosurgery, West China Hospital, Sichuan University, Chengdu, China

**Keywords:** sepsis, autoimmune diseases, Mendelian randomization, 28-day mortality, risk factor

## Abstract

**Background:**

Autoimmune diseases exhibit heterogenous dysregulation of pro-inflammatory or anti-inflammatory cytokine expression, akin to the pathophysiology of sepsis. It is speculated that individuals with autoimmune diseases may have an increased likelihood of developing sepsis and face elevated mortality risks following septic events. However, current observational studies have not yielded consistent conclusions. This study aims to explore the causal relationship between autoimmune diseases and the risks of sepsis and mortality using Mendelian randomization (MR) analysis.

**Methods:**

We conducted a two-sample MR study involving a European population, with 30 autoimmune diseases as the exposure factors. To assess causal relationships, we employed the inverse variance-weighted (IVW) method and used Cochran's Q test for heterogeneity, as well as the MR pleiotropy residual sum and outlier (MR-PRESSO) global test for potential horizontal pleiotropy.

**Results:**

Genetically predicted Crohn's disease (*β* = 0.067, se = 0.034, *p* = 0.046, OR = 1.069, 95% CI = 1.001–1.141) and idiopathic thrombocytopenic (*β* = 0.069, se = 0.031, *p* = 0.023, OR = 1.071, 95% CI = 1.009–1.136) were positively associated with an increased risk of sepsis in critical care. Conversely, rheumatoid arthritis (*β* = −0.104, se = 0.047, *p* = 0.025, OR = 0.901, 95% CI = 0.823–0.987), ulcerative colitis (*β* = −0.208, se = 0.084, *p* = 0.013, OR = 0.812, 95% CI = 0.690–0.957), and narcolepsy (*β* = −0.202, se = 0.092, *p* = 0.028, OR = 0.818, 95% CI = 0.684–0.978) were associated with a reduced risk of sepsis in critical care. Moreover, Crohn's disease (*β* = 0.234, se = 0.067, *p* = 0.001, OR = 1.263, 95% CI = 1.108–1.440) and idiopathic thrombocytopenic (*β* = 0.158, se = 0.061, *p* = 0.009, OR = 1.171, 95% CI = 1.041–1.317) were also linked to an increased risk of 28-day mortality of sepsis in critical care. In contrast, multiple sclerosis (*β* = −0.261, se = 0.112, *p* = 0.020, OR = 0.771, 95% CI = 0.619–0.960) and narcolepsy (*β* = −0.536, se = 0.184, *p* = 0.003, OR = 0.585, 95% CI = 0.408–0.838) were linked to a decreased risk of 28-day mortality of sepsis in critical care.

**Conclusion:**

This MR study identified causal associations between certain autoimmune diseases and risks of sepsis in critical care, and 28-day mortality in the European population. These findings suggest that exploring the mechanisms underlying autoimmune diseases may offer new diagnostic and therapeutic strategies for sepsis prevention and treatment.

## Introduction

Sepsis is characterized as a severe whole-body inflammation in response to infection, which has the potential to cause organ dysfunction and mortality (1). It is regarded as one of the “oldest and most elusive syndromes in medicine” (2). The annual incidence of sepsis is approximately 437 cases per 100,000 people, with over half of the patients experiencing severe sepsis, resulting in approximately 5.3 million deaths annually (3). Intensive care unit (ICU) admission is frequently caused by severe sepsis, resulting in an approximate mortality rate of 30% (4, 5).

The pathogenesis of sepsis is related to dysregulated immune responses triggered by pathogen invasion, leading to sustained and excessive inflammation and immunosuppression (6). This hyperactive inflammatory response is considered a key driving factor behind sepsis-related mortality, thus garnering significant attention in recent years regarding the dysregulation of pro-inflammatory and anti-inflammatory pathways (7–9).

Autoimmune diseases consist of a wide range of disorders characterized by aberrant immune reactions of hyperactive immune cells against the body's healthy tissues. In certain autoimmune diseases, there exists dysregulation of cytokine expression pathways similar to those seen in sepsis pathophysiology (6, 10, 11). Alterations in cytokine levels can influence the likelihood and consequences of sepsis in autoimmune individuals, making it crucial to investigate the variation in cytokine levels at different stages of sepsis development in individuals with autoimmune diseases (12–14).

Based on previous research, individuals with autoimmune diseases may face a higher likelihood of sepsis occurrence and post-sepsis mortality due to the immune dysfunction they experience (15, 16). However, a recent observational study conducted this year has yielded contrary conclusions. It suggests a protective association between certain autoimmune diseases with sepsis occurrence and mortality (17). Given the high heterogeneity of both autoimmune diseases and sepsis, factors such as the use of immunosuppressants, corticosteroids, and other confounding variables have led to contradictory findings in different observational studies (17–19). Hence, it is essential to employ an accurate and persuasive method to analyze the relationship between these two conditions. In this context, MR using single nucleotide polymorphisms (SNPs) as instrumental variables (IVs) provides an effective means to explore causal relationships between exposures and outcomes. The fundamental assumption of MR study is that traits determined by genes are less susceptible to measurement errors or confounding influences. Compared to traditional observational studies, MR study is less affected by measurement errors or confounding and are less prone to reverse causality; thus, it is recommended for research on sepsis (20–22).

This study employs two-sample MR analysis to assess the association between genetically predicted autoimmune diseases and the risk of sepsis in critical care and 28-day mortality. We identified 30 autoimmune diseases with available genetic instruments and evaluated the risks of two outcomes: sepsis in critical care and sepsis 28-day mortality in critical care.

## Methods

### Study design

**Figure 1:**
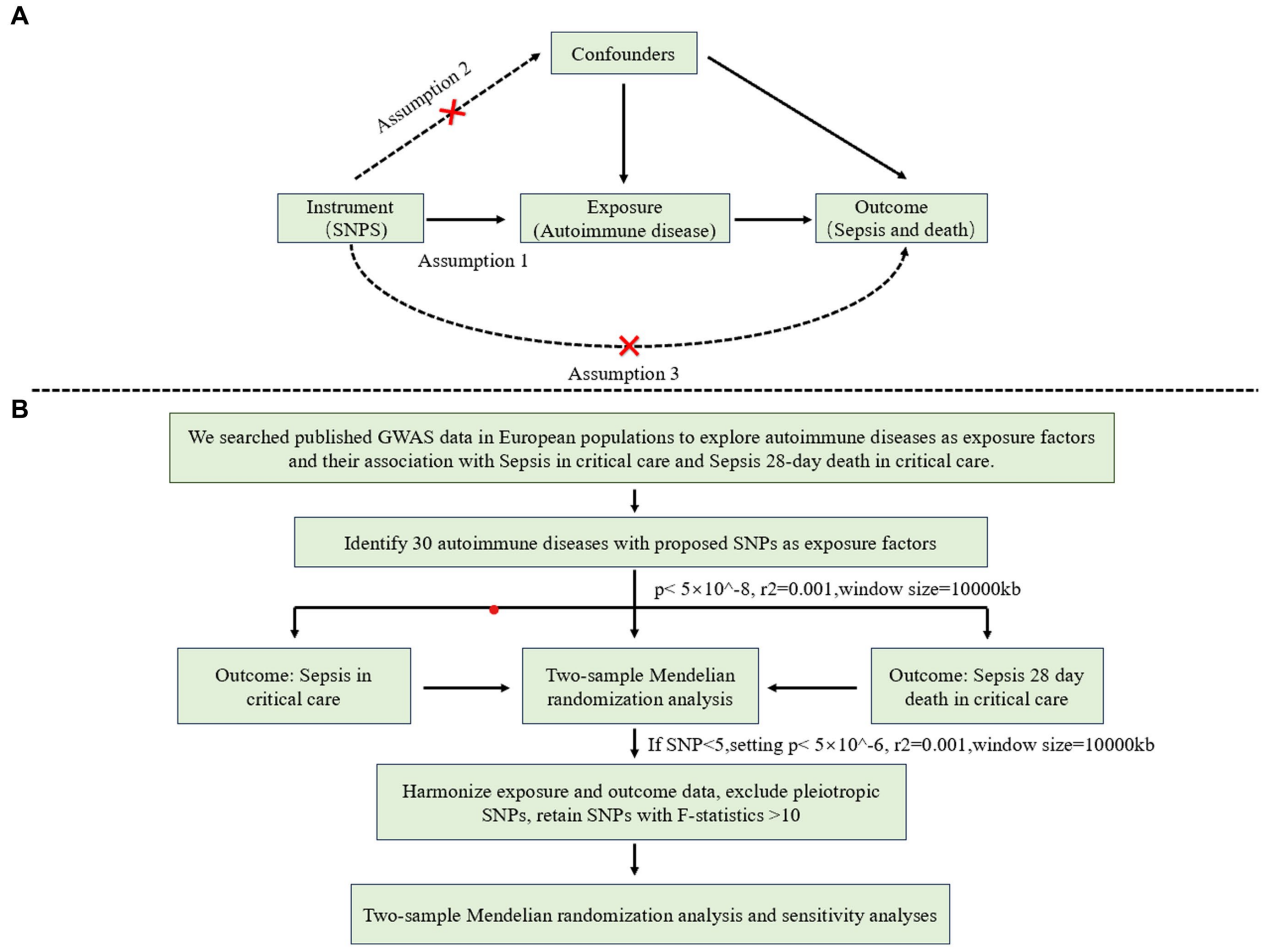
**(A)** The design of summary-data-based Mendelian randomization (SMR) model, MR analysis must satisfy three key assumptions: 1. Genetic variables are significantly associated with exposure; 2. Genetic variation serving as IVs for exposure is unrelated to other confounding factors; 3. Genetic variation affects the outcome solely through its impact on the exposure (without pleiotropic effects). **(B)** Flow-chart of study design, illustrates the step-by-step process of conducting genetic data sources and instruments the MR analysis.

This study follows the reporting guidelines of STROBE-MR (23). MR analysis must satisfy three key assumptions (Figure 1): 1. Genetic variables are significantly associated with exposure; 2. Genetic variation serving as IVs for exposure is unrelated to other confounding factors; 3. Genetic variation affects the outcome solely through its impact on the exposure (without pleiotropic effects). Figure 1B illustrates a summary of the study design.

First, we searched through the open-access summary genetic data in the IEU OpenGWAS Project,^1^ encompassing all available autoimmune diseases as exposure factors and sepsis in critical care and sepsis 28-day mortality in critical care as outcomes. The Single nucleotide polymorphisms (SNPs) were selected as the instrumental variables (IVs), where casual effects between exposures and outcomes were estimated with two-sample MR analysis. The diagnoses of diseases were further confirmed with the standard codes, the International Classification of Diseases, 10th revision (ICD-10), which were provided in the GWAS dataset. The study did not establish a prospective plan or disclose an analysis plan.

### Sepsis GWAS data

The summary genetic data of sepsis in critical care and sepsis 28-day mortality in critical care were extracted from the UK Biobank, a substantial cohort of adult volunteers in the UK with all participants providing written informed consent for research participation (24). A total number of 1380 cases were identified with sepsis in the ICU, compared with 429,985 control cases. Approximately 347 patients died within 28 days of critical care admission. Detailed information is provided in Supplementary Tables S1, S2.

### Autoimmune disease GWAS data

The open-access summary genetic data of autoimmune diseases were downloaded from the in individuals of European descent in GWAS catalogs and PubMed. After reviewing recruitment procedures and diagnostic criteria, we excluded trials with potential significant overlap between GWAS populations and selected a total of 30 autoimmune diseases representing 7 types as exposures. Diagnosis of autoimmune diseases were confirmed with the provided ICD codes, where the original GWAS researches (25–31) and detailed information are summarized in Supplementary Tables S1, S2.

### Selection of genetic IVs

In the selection of genetic IVs, we employed two strategies to ensure the accuracy of the experiment. First, we extracted IVs for the exposure factors (autoimmune diseases) with a genome-wide significance threshold of *p* < 5 × 10^−8^. In this step, if the number of SNPs extracted for a particular autoimmune disease was less than 5, we adjusted the significance level to *p* < 5 × 10^−6^ to include more SNPs as IVs and reduce potential errors caused by limited IVs. The extracted SNPs are summarized (Supplementary Table S3). To ensure independence between SNPs, we pruned the SNPs in the exposure factors using a threshold of *r*^2^ > 0.001 and a data distance of 10,000 kb to eliminate the impact of linkage disequilibrium (LD). An allele frequency threshold (MAF) of 0.3, which allowed the presence of palindromic SNPs, was applied to the remaining SNPs. Finally, we harmonized the genetic data to ensure the causal effects of SNPs on the exposure factors matched the same alleles affecting each outcome.

### Statistical analysis

The reliable interpretation of causal estimation in MR analysis depends on meeting the three key assumptions mentioned earlier. Heterogeneity in causal estimates among IVs indicates potential violations of the MR analysis assumptions (32). The research utilized two-sample Mendelian randomization analysis. In order to confirm assumption 1, we computed the F value, which measures the strength of the association between SNPs and the exposure (*F* = bet^2^/se^2^) (33). To avoid weak instrument bias, we kept SNPs with an F statistic exceeding 10. The retained SNPs were then used as IVs in subsequent analyses to address the causal analysis regarding whether the exposure factors influence the outcomes. In the study, we utilized the inverse variance-weighted (IVW) method (fixed/random effects) as the primary analysis. The IVW method precisely meta-analyzes the specific effects of exposure on each SNP (34). We performed Cochran's Q test for potential heterogeneity. The MR pleiotropy residual sum and outlier (MR-PRESSO) global test and MR Egger test (MR Egger intercept) were conducted to identify any potential balanced or imbalanced pleiotropy (35). If a set of SNPs exhibited heterogeneity, subsequent analyses employed the random-effects IVW method, while SNPs without heterogeneity were analyzed using the fixed-effects IVW method. As the exchangeability and exclusion restriction assumptions cannot be formally tested, we conducted extensive sensitivity analyses using the MR Egger method (36), the weighted median method (37), the weighted mode method (38), and the MR-PRESSO outlier correction test (35), comparing their results with the IVW (fixed/random effects) to estimate the causal relationship of the core assumption.

All effect size and standard error calculations for all results and the calculation of the odds ratio (OR) with 95% confidence intervals for binary outcomes were performed using the “TwoSampleMR,” “mendelianrandomization,” and “MR-PRESSO” packages in R (version 4.2.1).

## Results

This study included 30 autoimmune diseases and 2 outcomes, providing specific information and diagnostic codes in Supplementary Table S1. Supplementary Table S2 summarizes detailed information on the included genome-wide analysis studies, and additional information about each disease can be found on the website (IEU OpenGWAS project at mrcieu.ac.uk) using the GWAS ID. We applied strict selection criteria and ultimately included 20 autoimmune diseases, with a genome-wide significance threshold of *p* < 5 × 10^−6^, while the remaining 10 autoimmune diseases had an even stricter genome-wide significance threshold of *p* < 5 × 10^−8^. All IVs had an F statistic greater than 10, indicating no evidence of weak instrument bias (Supplementary Table S3).

### Risk of autoimmune diseases for sepsis in critical care

**Figure 2:**
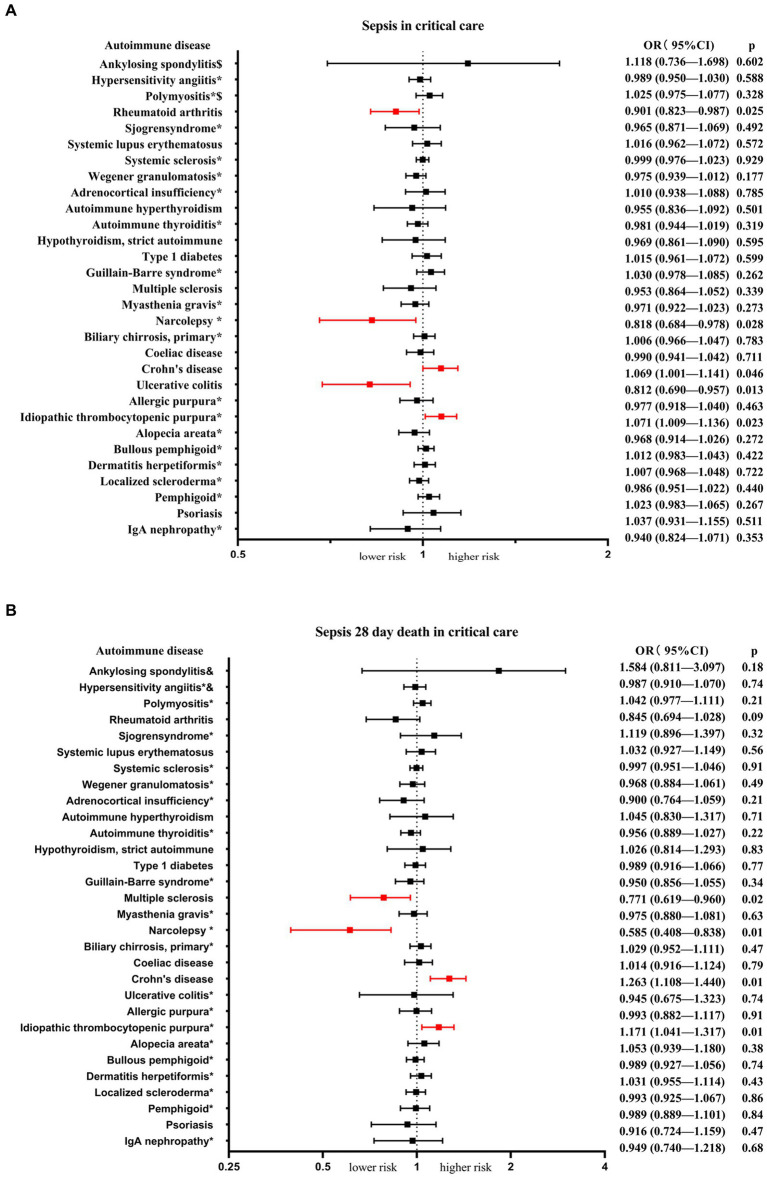
The MR analysis presents the causal estimations of 30 autoimmune diseases on the sepsis in critical care and sepsis 28-day mortality in critical care. **(A)** Exhibits the causal effects between the 30 autoimmune diseases and sepsis in critical care. **(B)** Illustrates the causal effects between the 30 autoimmune diseases and sepsis 28-day mortality in critical care. The odds ratios (ORs) were estimated using the IVW method, with the horizontal bars denoting the 95% confidence intervals (CIs).

Among the 30 autoimmune diseases considered as exposure factors, 5 of them showed statistically significant associations with an increased or decreased risk of sepsis in critical care (Table 1; Figure 2A). There was no apparent causal relationship between the remaining 25 autoimmune diseases and sepsis in critical care. IVW analysis revealed that Crohn's disease (*β* = 0.067, se = 0.034, *p* = 0.046, OR = 1.069, 95% CI = 1.001–1.141) and idiopathic thrombocytopenic (*β* = 0.069, se = 0.031, *p* = 0.023, OR = 1.071, 95% CI = 1.009–1.136) were associated with an increased risk of sepsis in critical care. On the other hand, rheumatoid arthritis (*β* = −0.104, se = 0.047, *p* = 0.025, OR = 0.901, 95% CI = 0.823–0.987), ulcerative colitis (*β* = −0.208, se = 0.084, *p* = 0.013, OR = 0.812, 95% CI = 0.690–0.957), and narcolepsy (*β* = −0.202, se = 0.092, *p* = 0.028, OR = 0.818, 95% CI = 0.684–0.978) were associated with a reduced risk of sepsis in critical care. Scatter plots, forest plots, leave-one-out plots, and funnel plots were generated to illustrate the specific effects and influences of SNPs on exposure and outcomes (Supplementary Figures S1–S5).

**Table 1:** An overview of the genetic instruments used in the MR study and the causal relationship between autoimmune disease and sepsis in critical care estimated by the inverse-variance weighted method.

Risk factor	Used SNPs^a^ SNPs	Sample	(b/se)	IVW P	IVW het P	Intercept P	MPO P
1. Connective tissue disease							
Ankylosing spondylitis^&^	25/26	22,647	0.112/0.214	0.602	0.038#	0.552	0.059
Hypersensitivity angiitis*	5/5	213,230	−0.012/0.021	0.588	0.819	0.570	0.776
Polymyositis*^&^	7/7	213,264	0.025/0.026	0.328	0.027#	0.086	0.069
Rheumatoid arthritis	10/10	153,457	−0.104/0.047	0.025#	0.521	0.900	0.579
Sjogrensyndrome*	12/13	214,435	−0.036/0.053	0.492	0.230	0.118	0.286
Systemic lupus erythematosus	36/43	14,267	0.016/0.028	0.572	0.110	0.748	0.110
Systemic sclerosis*	6/7	218,606	−0.002/0.013	0.929	0.464	0.830	0.630
Wegener granulomatosis*	8/9	213,388	−0.026/0.02	0.177	0.510	0.725	0.595
2. Endocrine system disease							
Adrenocortical insufficiency*	9/10	211,526	0.011/0.038	0.785	0.188	0.986	0.226
Autoimmune hyperthyroidism	5/5	173,938	−0.046/0.069	0.501	0.062	0.926	0.168
Autoimmune thyroiditis*	9/11	187,928	−0.02/0.02	0.319	0.333	0.598	0.417
Hypothyroidism, strict autoimmune	41/56	198,472	−0.032/0.061	0.595	0.403	0.593	0.372
Type 1 diabetes	7/8	185,115	0.015/0.028	0.599	0.052	0.552	0.148
3. Neurological disease							
Guillain-Barre syndrome*	6/6	215,931	0.03/0.027	0.262	0.884	0.879	0.934
Multiple sclerosis	25/26	27,098	−0.048/0.051	0.339	0.444	0.651	0.505
Myasthenia gravis*	8/8	217,288	−0.03/0.027	0.273	0.954	0.928	0.966
Narcolepsy*	5/5	12,307	−0.202/0.092	0.028#	0.970	0.627	0.971
4. Digestive disease							
Biliary cirrhosis, primary*	12/12	176,861	0.006/0.021	0.783	0.378	0.194	0.374
Coeliac disease	8/8	212,937	−0.01/0.027	0.711	0.558	0.408	0.626
Crohn's disease*	104/115	51,874	0.067/0.034	0.046#	0.807	0.701	0.813
Ulcerative colitis*	5/6	212,551	−0.208/0.084	0.013#	0.833	0.683	0.866
5. Hematologic disease							
Allergic purpura*	12/12	216,569	−0.024/0.032	0.463	0.339	0.235	0.388
Idiopathic thrombocytopenic purpura*	11/11	216,493	0.069/0.031	0.023#	0.966	0.999	0.984
6. Dermatology							
Alopecia areata*	8/12	211,428	−0.033/0.03	0.272	0.831	0.798	0.862
Bullous pemphigoid*	12/14	218,285	0.013/0.016	0.422	0.515	0.187	0.511
Dermatitis herpetiformis*	12/14	218,344	0.008/0.021	0.722	0.158	0.993	0.253
Localized scleroderma*	4/4	207,662	−0.015/0.019	0.440	0.735	0.830	0.766
Pemphigoid*	9/12	218,348	0.023/0.021	0.267	0.480	0.987	0.447
Psoriasis	11/13	216,752	0.037/0.056	0.511	0.358	0.168	0.412
7. Urologic disease							
IgA nephropathy*	4/4	5,957	−0.063/0.067	0.353	0.081	0.191	0.176

MR, Mendelian randomization; SNP, single nucleotide polymorphism; IVW, inverse-variance weighted; b, Beta; se, standard error; het, heterogeneity; MPO, MR-PRESSO. *Genome-wide significance of the selected SNPs associated with the factors is less than 5 × 10^−6^. ^a^SNPs used in the present MR analysis. ^#^*p* < 0.05. ^&^Uesd IVW random effect model.

In the sensitivity analysis, we performed random-effects IVW analysis for ankylosing spondylitis and polymyositis to assess heterogeneity, and the results showed that the robustness of the findings remained unchanged. Based on the results of MR-PRESSO global test, pleiotropic effects (*p* > 0.05) was not presented in the exposures. Among the autoimmune diseases that showed statistically significant associations in the IVW analysis, the results of MR–Egger analysis, weighted median method analysis, and weighted mode method analysis were consistent with the main analysis direction, demonstrating the robustness of the results (Supplementary Table S4-1).

### Risk of autoimmune diseases for sepsis 28-day mortality in critical care

Next, we conducted an analysis of the risk of sepsis 28-day mortality in critical care associated with autoimmune diseases. The results revealed that 4 autoimmune diseases showed statistically significant associations with an increased or decreased risk of sepsis 28-day mortality in critical care (Table 2; Figure 2B). IVW analysis indicated that Crohn's disease (*β* = 0.234, se = 0.067, *p* = 0.001, OR = 1.263, 95% CI = 1.108–1.440) and idiopathic thrombocytopenic (*β* = 0.158, se = 0.061, *p* = 0.009, OR = 1.171, 95% CI = 1.041–1.317) were associated with an increased risk of sepsis 28-day mortality in critical care. On the other hand, systemic sclerosis (*β* = −0.261, se = 0.112, *p* = 0.020, OR = 0.771, 95% CI = 0.619–0.960) and narcolepsy (*β* = −0.536, se = 0.184, *p* = 0.003, OR = 0.585, 95% CI = 0.408–0.838) were associated with a reduced risk of sepsis 28-day mortality in critical care. Scatter plots, forest plots, leave-one-out plots, and funnel plots were generated to illustrate the specific effects and influences of SNPs on exposure and outcomes (Supplementary Figures S6–S9).

**Table 2:** An overview of the genetic instruments used in the MR study and the causal relationship between autoimmune disease and 28 day death in critical care estimated by the inverse-variance weighted method.

Risk factor	Used SNPs^a^ SNPs	Sample	(b/se)	IVW P	IVW het P	Intercept P	MPO P
1. Connective tissue disease							
Ankylosing spondylitis	25/26	22,647	0.461/0.342	0.178	0.742	0.444	0.773
Hypersensitivity angiitis*	5/5	213,230	−0.014/0.042	0.743	0.912	0.559	0.914
Polymyositis*	7/7	213,264	0.042/0.033	0.207	0.478	0.498	0.437
Rheumatoid arthritis	10/10	153,457	−0.169/0.101	0.092	0.311	0.375	0.370
Sjogrensyndrome*	12/13	214,435	0.113/0.114	0.321	0.129	0.069	0.163
Systemic lupus erythematosus	36/43	14,267	0.032/0.055	0.561	0.117	0.466	0.132
Systemic sclerosis*	6/7	218,606	−0.003/0.025	0.909	0.675	0.369	0.671
Wegener granulomatosis*	8/9	213,388	−0.033/0.047	0.488	0.169	0.052	0.217
2. Endocrine system disease							
Adrenocortical insufficiency*	9/10	211,526	−0.106/0.084	0.205	0.090	0.249	0.114
Autoimmune hyperthyroidism	5/5	173,938	0.045/0.118	0.706	0.157	0.807	0.338
Autoimmune thyroiditis*	9/11	187,928	−0.046/0.037	0.221	0.863	0.681	0.868
Hypothyroidism, strict autoimmune	41/56	198,472	0.026/0.119	0.827	0.785	0.294	0.775
Type 1 diabetes	7/8	185,115	−0.012/0.039	0.767	0.568	0.348	0.541
3. Neurological disease							
Guillain-Barre syndrome*	6/6	215,931	−0.051/0.054	0.339	0.409	0.329	0.398
Multiple sclerosis	25/26	27,098	−0.261/0.112	0.020#	0.180	0.563	0.228
Myasthenia gravis*	8/8	217,288	−0.026/0.053	0.633	0.700	0.960	0.791
Narcolepsy*	5/5	12,307	−0.536/0.184	0.003#	0.757	0.455	0.781
4. Digestive disease							
Biliary cirrhosis, primary*	12/12	176,861	0.029/0.04	0.472	0.627	0.203	0.601
Coeliac disease	8/8	212,937	0.015/0.053	0.787	0.904	0.631	0.904
Crohn's disease	104/115	51,874	0.234/0.067	0.001#	0.809	0.436	0.821
Ulcerative colitis*	5/6	212,551	−0.057/0.172	0.741	0.377	0.996	0.433
5. Hematologic disease							
Allergic purpura*	12/12	216,569	−0.008/0.061	0.907	0.814	0.740	0.812
Idiopathic thrombocytopenic purpura*	11/11	216,493	0.158/0.061	0.009#	0.825	0.712	0.854
6. Dermatology							
Alopecia areata*	8/12	211,428	0.052/0.059	0.377	0.525	0.329	0.535
Bullous pemphigoid*	12/14	218,285	−0.011/0.034	0.744	0.298	0.665	0.316
Dermatitis herpetiformis*	12/14	218,344	0.031/0.04	0.430	0.211	0.131	0.283
Localized scleroderma*	4/4	207,662	−0.007/0.037	0.856	0.917	0.614	0.794
Pemphigoid*	9/12	218,348	−0.011/0.055	0.844	0.074	0.359	0.094
Psoriasis	11/13	216,752	−0.088/0.121	0.466	0.221	0.902	0.338
7. Urologic disease							
IgA nephropathy*	4/4	5957	−0.052/0.128	0.683	0.111	0.158	0.204

MR, Mendelian randomization; SNP, single nucleotide polymorphism; IVW, inverse-variance weighted; b, Beta; se, standard error; het, heterogeneity; MPO, MR-PRESSO. *Genome-wide significance of the selected SNPs associated with the factors is less than 5 × 10^−6^. ^a^SNPs used in the present MR analysis. ^#^*p* < 0.05.

In the sensitivity analysis, no evidence of heterogeneity or pleiotropy was found. Among the four autoimmune diseases that showed statistically significant associations in the IVW analysis, the results of MR–Egger analysis, weighted median method analysis, and weighted mode method analysis were consistent with the main analysis direction (Supplementary Table S4-2).

## Discussion

In this study, we conducted a two-sample MR research aimed at exploring the relationship between 30 autoimmune diseases and the occurrence of sepsis leading to ICU admission, as well as the 28-day mortality rate among those admitted to the ICU with sepsis. In comparison to previous observational studies, our MR findings presented both congruent and distinctive conclusions, which were meticulously elucidated through in-depth analysis.

We revealed causal associations between three autoimmune diseases and the two aforementioned outcomes, while another three autoimmune diseases exhibited causal links with individual outcomes. Specifically, Crohn's disease and idiopathic thrombocytopenia were established as risk factors for sepsis in critical care and sepsis 28-day mortality in critical care, respectively. Conversely, narcolepsy demonstrated a protective association with both sepsis in critical care and sepsis 28-day mortality in critical care. Rheumatoid arthritis and ulcerative colitis were identified as protective factors against sepsis in critical care. Additionally, systemic sclerosis exhibited a protective effect on sepsis 28-day mortality in critical care. These findings further enrich our comprehension of the interplay between autoimmune diseases and severe sepsis while also facilitating a deeper exploration of the intricate interrelationships between inflammation and septic conditions (39).

In previous studies, it has been observed that platelets are the primary effectors of inflammation and hemostasis and may exacerbate the dysregulated host response during sepsis, thereby increasing the risk of severe sepsis and mortality (40–42). Consistent with our findings, our study corroborates that idiopathic thrombocytopenia is a risk factor for sepsis in critical care and sepsis 28-day mortality in critical care. Immunothrombosis is a protective response that occurs when pathogens infiltrate the human body, triggering the activation of the coagulation system and causing microvascular thrombosis in the vicinity. This defense mechanism confines the infection to the specific region. However, when idiopathic thrombocytopenia is present, this defensive mechanism is weakened, thereby elevating the risk of severe sepsis occurrence (43).

Surprisingly, our study revealed opposing effects of Crohn's disease and ulcerative colitis on sepsis. Crohn's disease emerged as a risk factor for sepsis in critical care and 28-day mortality, while ulcerative colitis exhibited a protective association with sepsis in critical care. This conclusion aligns with a previous sepsis and autoimmune disease cohort study using the MIMIC III database but differs from that study in terms of the statistically insignificant reduction in 30-day mortality risk associated with ulcerative colitis (OR=0.87, 95% CI = 0.52–1.43, *P* = 0.594) (17). A similar study using the US national inpatient data arrived at opposing conclusions, showing a statistically significant decrease in the risk of death associated with Crohn's disease (OR = 0.78, 95% CI = 0.63–0.97) and a statistically significant increase in mortality risk associated with ulcerative colitis (OR = 1.61, 95% CI = 1.35–1.93) (19). However, these observational studies did not account for the potential impact of treatment differences between the two diseases. In fact, Crohn's disease patients are 5–10 times more likely to receive anti-tumor necrosis factor-α (TNF-α) treatment than ulcerative colitis patients, which could be a key influencing factor leading to differing results between previous studies and our findings (44, 45). The opposing effects of Crohn's disease and ulcerative colitis on outcomes can be explained by the primary sites of inflammation in the two diseases: inflammation in ulcerative colitis is predominantly limited to the intestinal mucosa, while transmural inflammation occurs primarily in Crohn's disease (46, 47). Moreover, these two diseases exhibit significant differences in other aspects as well. Further research into the mechanisms of inflammation between Crohn's disease and ulcerative colitis may provide new insights into the inflammatory response to sepsis (48, 49).

Regarding the impact of rheumatoid arthritis on sepsis, previous research has shown some controversy. in Germany, A retrospective study conducted suggested an independent correlation between rheumatoid arthritis and increased sepsis mortality (50). Another retrospective study found that rheumatoid arthritis was a significant independent risk factor for increased long-term mortality in sepsis patients (OR: 1.63, 95% CI: 1.03–1.63, *p* = 0.04), but it did not have an independent effect on short-term mortality risk after admission (51). Conversely, two other studies considered rheumatoid arthritis as a protective factor against short-term mortality in sepsis patients (17, 52). In our MR study, we concluded that rheumatoid arthritis is a protective factor for sepsis in critical care, but it is not associated with changes in short-term mortality risk. The underlying reason for this phenomenon might be the overexpression of cytokines IL-12 and IFN-γ in rheumatoid arthritis patients, with relative deficiencies of IL-4 and IL-10 (53). Studies have suggested that therapies increasing the expression of IL-12 and IFN-γ can improve sepsis survival rates (54). We hypothesize that the overexpression of certain cytokines in rheumatoid arthritis patients may reduce the likelihood of immune dysfunction, thereby decreasing the risk of severe sepsis occurrence (17, 55).

Among the remaining autoimmune diseases with causal relationships to sepsis, only a few retrospective studies are available for reference. As mentioned earlier, a cohort study on sepsis and autoimmune diseases using the MIMIC III database found that multiple sclerosis is a protective factor for sepsis mortality (HR: 0.45, 95% CI: 0.22–0.89, *p* = 0.023), which aligns with our conclusion (17). This study proposed that multiple sclerosis patients with specific cytokine overexpression or deficiencies before sepsis may be more likely to survive when immune function is compromised (56–58). Narcolepsy, a chronic sleep disorder, is caused by the depletion of a small number of hypothalamic neurons responsible for generating neuropeptides that promote wakefulness (59). We found that narcolepsy is a protective factor for severe sepsis and 28-day mortality in ICU sepsis patients, but this result has not been confirmed by existing observational studies. According to previous research, narcolepsy patients tend to secrete higher levels of cytokines, including IL-2, tumor necrosis factor, IL-4, and IL-13, which could be the reason for narcolepsy being a protective factor against severe sepsis and 28-day mortality in sepsis patients (60, 61).

In this study, we conducted a two-sample MR study to comprehensively assess the causal relationships between 30 autoimmune diseases and the risk of sepsis in critical care and sepsis 28-day mortality in critical care. Unlike previous observational studies, both sepsis and autoimmune diseases are highly heterogeneous, making it challenging to avoid confounding factors (20). Due to the uniqueness of autoimmune diseases and sepsis, conducting randomized controlled trials would be prohibitively time consuming and costly. Therefore, our MR design is less susceptible to measurement errors, confounding, and reverse causation compared to traditional observational studies, enabling us to better reveal causal relationships. These findings are crucial for a deeper understanding of the association between autoimmune diseases and severe sepsis, as well as for further exploring the interplay between inflammation and sepsis (62).

There are certain limitations in our research. First, the focus of this research was on European populations, and the restriction to a specific ethnicity may impact the generalizability of the results to other ethnic groups. Second, due to the limited availability of open-access genetic data on autoimmune diseases, we slightly relaxed the genome-wide significance threshold (*p* < 5 × 10^−6^) in some exposure factors, which might influence our interpretation of causal relationships between certain genes and autoimmune diseases. Additionally, the accuracy of interpreting causal relationships using genetic instruments is limited and cannot completely eliminate the influence of all confounding factors. Hence, cautious interpretation of the results is necessary to avoid overinterpretation. Last, our study results provide genetic evidence for the causal impact of autoimmune diseases on the risk of severe sepsis and 28-day mortality, but it does not delve into detailed mechanistic explanations. Therefore, in future research, further exploration of the specific biological mechanisms between autoimmune diseases and sepsis is needed. This includes studying the differences in inflammation and immune pathways among autoimmune disease patients and how these differences influence the onset and prognosis of sepsis. Additionally, factors such as age, gender and the specific impact of immunosuppressants require more real-world studies. Through such research, we may discover new therapeutic strategies and novel insights into the prevention and treatment of sepsis.

In conclusion, this MR study identified causal associations between certain autoimmune diseases and risks of sepsis in critical care and 28-day mortality in the European population. The findings contribute robust evidence, advancing our comprehension of the intricate relationship between autoimmune diseases and severe sepsis. The identification of these causal associations suggests that delving into autoimmune disease-related mechanisms could potentially unveil novel therapeutic strategies for the prevention and treatment of sepsis. Nonetheless, further research is required to validate the findings and to reveal the underlying clinical mechanism.

## Data availability statement

The original contributions presented in the study are included in the article/Supplementary material, further inquiries can be directed to the corresponding author.

## Author contributions

XT: Data curation, Formal analysis, Project administration, Resources, Validation, Visualization, Writing – original draft, Writing – review & editing. YZ: Data curation, Formal analysis, Project administration, Resources, Software, Visualization, Writing – review & editing. JS: Funding acquisition, Investigation, Software, Writing – review & editing. XL: Investigation, Methodology, Project administration, Writing – review & editing. TZ: Conceptualization, Validation, Writing – review & editing. WY: Writing – review & editing.

## Footnotes

^1^ https://gwas.mrcieu.ac.uk/
