# Supplementary material for: Genetic associations between autoimmune diseases and the risks of severe sepsis and 28-day mortality: a two-sample Mendelian randomization study
Source: Front Med (Lausanne). 2024 Jan 26;11:1331950. doi: 10.3389/fmed.2024.1331950 (PMC10853392; doi:10.3389/fmed.2024.1331950)
Supplement: Supplementary file 1 [file Data_Sheet_1.zip › List of Supplementary Materials.DOCX]

**List of supplementary materials**

| Code | Material |
| --- | --- |
| Supplementary table 1 | Check list |
| Supplementary table 2 | Overview of diagnostic criteria for disease-related exposures and outcomes |
| Supplementary table 3 | The sources and information of the GWAS data for all exposures and outcomes. |
| Supplementary table 4 | The 2-sample MR analyses results with statistical significance |
| Supplementary table 5 | Sensitivity analysis results of the Weighted median, Maximum likelihood, and MR Egger regression methods. |
| Supplementary figure 1 | A:Scatter plots, B:forest plots, C:leave-one-out plots and D:funnel plots of Rheumatoid arthritis in Sepsis (critical care) |
| Supplementary figure 2 | A:Scatter plots, B:forest plots, C:leave-one-out plots and D:funnel plots of Narcolepsy in Sepsis (critical care) |
| Supplementary figure 3 | A:Scatter plots, B:forest plots, C:leave-one-out plots and D:funnel plots of Crohn in Sepsis (critical care) |
| Supplementary figure 4 | A:Scatter plots, B:forest plots, C:leave-one-out plots and D:funnel plots of Ulcerative colitis in Sepsis (critical care) |
| Supplementary figure 5 | A:Scatter plots, B:forest plots, C:leave-one-out plots and D:funnel plots of Idiopathic thrombocytopenic purpura in Sepsis (critical care) |
| Supplementary figure 6 | A:Scatter plots, B:forest plots, C:leave-one-out plots and D:funnel plots of Multiple sclerosis in Sepsis (28 day death in critical care) |
| Supplementary figure 7 | A:Scatter plots, B:forest plots, C:leave-one-out plots and D:funnel plots of Narcolepsy in Sepsis (28 day death in critical care) |
| Supplementary figure 8 | A:Scatter plots, B:forest plots, C:leave-one-out plots and D:funnel plots of Crohn's disease in Sepsis (28 day death in critical care) |
| Supplementary figure 9 | A:Scatter plots, B:forest plots, C:leave-one-out plots and D:funnel plots of Idiopathic thrombocytopenic purpura in Sepsis (28 day death in critical care) |
