# Supplementary material for: Genetic associations between autoimmune diseases and the risks of severe sepsis and 28-day mortality: a two-sample Mendelian randomization study
Source: Front Med (Lausanne). 2024 Jan 26;11:1331950. doi: 10.3389/fmed.2024.1331950 (PMC10853392; doi:10.3389/fmed.2024.1331950)
Supplement: Supplementary file 1 [file Data_Sheet_1.zip › Table 1.DOCX]

**Supplementary table 1**

| **Overview of diagnostic criteria for disease-related exposures and outcomes** | |
| --- | --- |
| **Outcome** | **ICD diagnostic number** |
| Sepsis （In critical care） | ICD-10: A41\|R57.2\|R65 |
| Sepsis (28 day death in critical care) | ICD-10: A41\|R57.2\|R65 |
| **Exposure** |  |
| **1.Connective tissue disease** |  |
| Ankylosing spondylitis | ICD-10: M45 |
| Hypersensitivity angiitis | ICD-10: M31.0 |
| Polymyositis | ICD-10: M33.2 |
| Rheumatoid arthritis | ICD-10:M05\|M06 |
| Sjogren syndrome | ICD-10:M35 |
| Systemic lupus erythematosus | ICD-10:M32 |
| Systemic sclerosis | ICD-10:M34 |
| Wegener granulomatosis | ICD-10: M31.3 |
| **2. Endocrine system** |  |
| Adrenocortical insufficiency | ICD-10: E27.1\|E27.2\|E27.3#\|E27.4 |
| Autoimmune hyperthyroidism | ICD-10: E05.9 |
| Autoimmune thyroiditis | ICD-10: E06.3 |
| Hypothyroidism, strict autoimmune | ICD-10: E03.8 |
| Type 1 diabetes | ICD-10: E10 |
| **3.** **Nervous system** |  |
| Guillain-Barre syndrome | ICD-10: G61.0 |
| Multiple sclerosis | ICD-10: G35 |
| Myasthenia gravis | ICD-10: G70.0 |
| Narcolepsy | ICD-10: G47.4 |
| **4. Digestive system** |  |
| Biliary chirrosis, primary | ICD-10: K74.3 |
| Coeliac disease | ICD-10: K90.0 |
| Crohn's disease | ICD-10: K50 |
| Ulcerative colitis | ICD-10: K51 |
| **5.** **Hematologic disease** |  |
| Allergic purpura | ICD-10: D69.0 |
| Idiopathic thrombocytopenic purpura | ICD-10: D69.3 |
| **6.** **Dermatology** |  |
| Alopecia areata | ICD-10: L63 |
| Bullous pemphigoid | ICD-10: L12.0 |
| Dermatitis herpetiformis | ICD-10: L13.0 |
| Localized scleroderma | ICD-10: L94 |
| Pemphigoid | ICD-10: L12 |
| Psoriasis | ICD-10: L40 |
| **7.** **Urologic disease** |  |
| IgA nephropathy | ICD-10: N08.2 |
