# Supplementary material for: Genetic associations between autoimmune diseases and the risks of severe sepsis and 28-day mortality: a two-sample Mendelian randomization study
Source: Front Med (Lausanne). 2024 Jan 26;11:1331950. doi: 10.3389/fmed.2024.1331950 (PMC10853392; doi:10.3389/fmed.2024.1331950)
Supplement: Supplementary file 1 [file Data_Sheet_1.zip › Table 2.DOCX]

**Supplementary table 2**

（The sources and information of the GWAS data for all exposures and outcomes. The GWAS data can be searched through the public IEU open GWAS project by Traits or GWAS ID. Link: <https://gwas.mrcieu.ac.uk/>）

| **Traits** | **GWAS ID** | **Sample** | **Ancestry** | **Units** |
| --- | --- | --- | --- | --- |
| **Outcome** | | | | |
| Sepsis （In critical care） | ieu-b-4982 | 431365 | European | logOR |
| Sepsis (28 day death in critical care) | ieu-b-4981 | 431365 | European | logOR |
| **Exposure** | | | | |
| **1.Connective tissue disease** |  |  |  |  |
| Ankylosing spondylitis | ebi-a-GCST005529 | 22647 | European | logOR |
| Hypersensitivity angiitis | finn-b-M13_HYPERANG | 213230 | European | logOR |
| Polymyositis | finn-b-M13_POLYMYO | 213264 | European | logOR |
| Rheumatoid arthritis | finn-b-M13_RHEUMA | 153457 | European | logOR |
| Sjogren syndrome | finn-b-M13_SJOGREN | 214435 | European | logOR |
| Systemic lupus erythematosus | ebi-a-GCST003156 | 14,267 | European | logOR |
| Systemic sclerosis | finn-b-SYSTSCLE_STRICT | 218606 | European | logOR |
| Wegener granulomatosis | finn-b-M13_WEGENER | 213388 | European | logOR |
| **2.Endocrine system** |  |  |  |  |
| Adrenocortical insufficiency | finn-b-E4_ADDISON | 211526 | European | logOR |
| Autoimmune hyperthyroidism | finn-b-AUTOIMMUNE_HYPERTHYROIDISM | 173938 | European | logOR |
| Autoimmune thyroiditis | finn-b-E4_THYROIDITAUTOIM | 187928 | European | logOR |
| Hypothyroidism, strict autoimmune | finn-b-E4_HYTHY_AI_STRICT | 198472 | European | logOR |
| Type 1 diabetes | finn-b-T1D_STRICT | 185115 | European | logOR |
| **3.** **Nervous system** |  |  |  |  |
| Guillain-Barre syndrome | finn-b-G6_GUILBAR | 215931 | European | logOR |
| Multiple sclerosis | ieu-a-1024 | 27098 | European | log odds |
| Myasthenia gravis | finn-b-G6_MYASTHENIA | 217288 | European | logOR |
| Narcolepsy | ebi-a-GCST005522 | 12307 | European | logOR |
| **4.Digestive system** |  |  |  |  |
| Biliary chirrosis, primary | finn-b-CHIRBIL_PRIM | 176861 | European | logOR |
| Coeliac disease | finn-b-K11_COELIAC | 212937 | European | logOR |
| Crohn's disease | ieu-a-12 | 51874 | European | log odds |
| Ulcerative colitis | finn-b-K11_UC_NOCD | 212551 | European | logOR |
| **5. Hematologic disease** |  |  |  |  |
| Allergic purpura | finn-b-D3_ALLERGPURPURA | 216569 | European | logOR |
| Idiopathic thrombocytopenic purpura | finn-b-D3_ITP | 216493 | European | logOR |
| **6. Dermatology** |  |  |  |  |
| Alopecia areata | finn-b-L12_ALOPECAREATA | 211428 | European | logOR |
| Bullous pemphigoid | finn-b-L12_PEMPHIGOID_BULL | 218285 | European | logOR |
| Dermatitis herpetiformis | finn-b-L12_DERMATHERP | 218344 | European | logOR |
| Localized scleroderma | finn-b-L12_LOCALSCLERODERMA | 207662 | European | logOR |
| Pemphigoid | finn-b-L12_PEMPHIGOID | 218348 | European | logOR |
| Psoriasis | finn-b-L12_PSORIASIS | 216752 | European | logOR |
| **7. Urologic disease** |  |  |  |  |
| IgA nephropathy | ieu-a-1081 | 5957 | European | log odds |
