# Supplementary material for: Genetic associations between autoimmune diseases and the risks of severe sepsis and 28-day mortality: a two-sample Mendelian randomization study
Source: Front Med (Lausanne). 2024 Jan 26;11:1331950. doi: 10.3389/fmed.2024.1331950 (PMC10853392; doi:10.3389/fmed.2024.1331950)
Supplement: Supplementary file 1 [file Data_Sheet_1.zip › Table 4.DOCX]

**Supplementary table 4-1** (Sepsis in critical care)Sensitivity analysis results of the MR Egger regression,Weighted median, and Weighted mode methods

|  | **MR Egger** | **Weighted median** | **Weighted mode** | **MR Egger** | **Weighted** | **Weighted median** |
| --- | --- | --- | --- | --- | --- | --- |
|  | **regression** | **median** | **mode** | **regression** | **median** | **mode** |
| **Risk factor** | **(b/se)** | **(b/se)** | **(b/se)** | **P** | **P** | **P** |
| **1.Connective tissue disease** |  |  |  |  |  |  |
| Ankylosing spondylitis | 0.083/0.23 | -0.035/0.239 | -0.035/0.239 | 0.856 | 0.718 | 0.886 |
| Hypersensitivity angiitis* | 0.006/0.034 | -0.012/0.025 | 0.002/0.028 | 0.872 | 0.628 | 0.940 |
| Polymyositis* | -0.039/0.036 | 0.011/0.022 | 0.014/0.022 | 0.328 | 0.614 | 0.553 |
| Rheumatoid arthritis | -0.097/0.071 | -0.082/0.054 | -0.082/0.055 | 0.207 | 0.129 | 0.167 |
| Sjogren syndrome* | 0.108/0.097 | 0.012/0.062 | 0.006/0.068 | 0.292 | 0.852 | 0.926 |
| Systemic lupus erythematosus | 0/0.057 | 0.015/0.04 | 0.039/0.051 | 0.993 | 0.716 | 0.444 |
| Systemic sclerosis* | 0.001/0.016 | 0.001/0.014 | 0/0.014 | 0.948 | 0.950 | 0.997 |
| Wegener granulomatosis* | -0.034/0.029 | -0.014/0.025 | -0.02/0.025 | 0.288 | 0.574 | 0.464 |
| **2.Endocrine system** |  |  |  |  |  |  |
| Adrenocortical insufficiency* | 0.009/0.084 | -0.018/0.044 | -0.025/0.056 | 0.918 | 0.684 | 0.669 |
| Autoimmune hyperthyroidism | -0.027/0.2 | -0.013/0.054 | 0.001/0.06 | 0.900 | 0.813 | 0.982 |
| Autoimmune thyroiditis* | -0.007/0.03 | -0.017/0.025 | -0.02/0.028 | 0.810 | 0.494 | 0.486 |
| Hypothyroidism, strict autoimmune | -0.101/0.141 | -0.025/0.099 | 0.022/0.117 | 0.480 | 0.801 | 0.853 |
| Type 1 diabetes | 0.054/0.068 | 0.027/0.023 | 0.028/0.022 | 0.466 | 0.234 | 0.240 |
| **3. Nervous system** |  |  |  |  |  |  |
| Guillain-Barre syndrome* | 0.025/0.037 | 0.03/0.033 | 0.03/0.033 | 0.530 | 0.366 | 0.403 |
| Multiple sclerosis | -0.013/0.092 | -0.011/0.072 | 0.006/0.071 | 0.893 | 0.879 | 0.933 |
| Myasthenia gravis* | -0.027/0.037 | -0.025/0.034 | -0.023/0.032 | 0.497 | 0.466 | 0.500 |
| Narcolepsy * | -0.471/0.509 | -0.206/0.106 | -0.173/0.153 | 0.423 | 0.052 | 0.321 |
| **4. Digestive system** |  |  |  |  |  |  |
| Biliary chirrosis, primary* | -0.032/0.033 | 0.005/0.026 | -0.01/0.034 | 0.361 | 0.846 | 0.766 |
| Coeliac disease | 0.014/0.037 | -0.008/0.029 | -0.005/0.029 | 0.718 | 0.796 | 0.863 |
| Crohn's disease | 0.098/0.087 | 0.09/0.051 | 0.059/0.077 | 0.265 | 0.076 | 0.444 |
| Ulcerative colitis* | -0.331/0.286 | -0.191/0.098 | -0.166/0.124 | 0.331 | 0.051 | 0.252 |
| **5. Hematologic disease** |  |  |  |  |  |  |
| Allergic purpura* | -0.07/0.048 | -0.056/0.043 | -0.062/0.042 | 0.177 | 0.198 | 0.168 |
| Idiopathicthrombocytopenic purpura* | 0.068/0.06 | 0.067/0.037 | 0.063/0.046 | 0.281 | 0.068 | 0.199 |
| **6. Dermatology** |  |  |  |  |  |  |
| Alopecia areata* | -0.042/0.046 | -0.03/0.039 | -0.038/0.042 | 0.398 | 0.446 | 0.400 |
| Bullous pemphigoid* | -0.009/0.021 | -0.009/0.02 | -0.012/0.023 | 0.687 | 0.659 | 0.616 |
| Dermatitis herpetiformis* | 0.007/0.034 | 0.005/0.021 | 0.007/0.021 | 0.840 | 0.829 | 0.749 |
| Localized scleroderma* | -0.01/0.025 | -0.014/0.022 | -0.017/0.023 | 0.722 | 0.517 | 0.514 |
| Pemphigoid* | 0.022/0.041 | -0.008/0.029 | -0.027/0.05 | 0.607 | 0.788 | 0.607 |
| Psoriasis | 0.122/0.078 | 0.076/0.061 | 0.076/0.066 | 0.151 | 0.214 | 0.276 |
| **7. Urologic disease** |  |  |  |  |  |  |
| IgA nephropathy* | 0.399/0.242 | -0.027/0.06 | -0.005/0.073 | 0.240 | 0.655 | 0.952 |

Abbreviations: Mendelian randomization (MR); inverse-variance weighted (IVW); Beta (b); standard error (se);

^a^ Genome-wide significance of the selected SNPs associated with the factors is less than 5×10-8, factors with * is less than 5×10-8.

* P<0.05

** P<0.01

**Supplementary table 5-2**(Sepsis 28 day death in critical care )Sensitivity analysis results of the MR Egger regression,Weighted median, and Weighted mode methods

|  | **MR Egger** | **Weighted median** | **Weighted mode** | **MR Egger** | **Weighted** | **Weighted median** |
| --- | --- | --- | --- | --- | --- | --- |
|  | **regression** | **median** | **mode** | **regression** | **median** | **mode** |
| **Risk factor** | **(b/se)** | **(b/se)** | **(b/se)** | **P** | **P** | **P** |
| **1.Connective tissue disease** |  |  |  |  |  |  |
| Ankylosing spondylitis | 0.499/0.463 | 0.408/0.473 | 0.408/0.473 | 0.168 | 0.281 | 0.397 |
| Hypersensitivity angiitis* | -0.049/0.068 | -0.028/0.051 | -0.03/0.059 | 0.524 | 0.585 | 0.643 |
| Polymyositis* | 0.006/0.058 | 0.009/0.043 | -0.028/0.065 | 0.923 | 0.829 | 0.685 |
| Rheumatoid arthritis | -0.277/0.153 | -0.254/0.107 | -0.259/0.103 | 0.108 | 0.017# | 0.032 |
| Sjogren syndrome* | 0.466/0.2 | 0.105/0.132 | 0.194/0.14 | 0.042 | 0.424 | 0.195 |
| Systemic lupus erythematosus | -0.041/0.113 | 0.098/0.073 | 0.074/0.09 | 0.721 | 0.177 | 0.414 |
| Systemic sclerosis* | -0.021/0.03 | -0.012/0.03 | -0.01/0.029 | 0.525 | 0.696 | 0.744 |
| Wegener granulomatosis* | -0.136/0.058 | -0.045/0.055 | -0.076/0.059 | 0.056 | 0.409 | 0.244 |
| **2.Endocrine system** |  |  |  |  |  |  |
| Adrenocortical insufficiency* | 0.08/0.168 | -0.202/0.094 | -0.232/0.133 | 0.650 | 0.033# | 0.120 |
| Autoimmune hyperthyroidism | 0.128/0.342 | 0.071/0.101 | 0.046/0.1 | 0.733 | 0.481 | 0.668 |
| Autoimmune thyroiditis* | -0.028/0.054 | -0.01/0.05 | -0.001/0.057 | 0.616 | 0.841 | 0.988 |
| Hypothyroidism, strict autoimmune | -0.237/0.274 | -0.066/0.178 | -0.209/0.254 | 0.392 | 0.710 | 0.416 |
| Type 1 diabetes | 0.073/0.09 | -0.022/0.05 | 0/0.05 | 0.455 | 0.658 | 0.997 |
| **3.** **Nervous system** |  |  |  |  |  |  |
| Guillain-Barre syndrome* | -0.109/0.074 | -0.11/0.068 | -0.104/0.069 | 0.217 | 0.107 | 0.194 |
| Multiple sclerosis | -0.361/0.206 | -0.306/0.145 | -0.321/0.145 | 0.093# | 0.035# | 0.037# |
| Myasthenia gravis* | -0.022/0.073 | -0.037/0.062 | -0.034/0.066 | 0.768 | 0.545 | 0.618 |
| Narcolepsy * | 0.325/1.022 | -0.409/0.231 | -0.392/0.295 | 0.771 | 0.077 | 0.255 |
| **4.Digestive system** |  |  |  |  |  |  |
| Biliary chirrosis, primary* | -0.045/0.066 | 0.003/0.054 | -0.031/0.07 | 0.516 | 0.955 | 0.664 |
| Coeliac disease | 0.041/0.075 | 0.009/0.056 | 0.016/0.056 | 0.602 | 0.866 | 0.778 |
| Crohn's disease | 0.108/0.174 | 0.187/0.107 | 0.192/0.171 | 0.538 | 0.081 | 0.265 |
| Ulcerative colitis* | -0.061/0.681 | 0.083/0.213 | 0.142/0.265 | 0.935 | 0.699 | 0.619 |
| **5. Hematologic disease** |  |  |  |  |  |  |
| Allergic purpura* | -0.032/0.095 | -0.061/0.088 | -0.08/0.089 | 0.743 | 0.487 | 0.386 |
| Idiopathicthrombocytopenic purpura* | 0.197/0.119 | 0.164/0.082 | 0.185/0.107 | 0.132 | 0.047# | 0.115 |
| **6. Dermatology** |  |  |  |  |  |  |
| Alopecia areata* | 0.124/0.09 | 0.099/0.081 | 0.115/0.085 | 0.216 | 0.219 | 0.216 |
| Bullous pemphigoid* | -0.026/0.048 | -0.036/0.043 | -0.042/0.055 | 0.603 | 0.400 | 0.459 |
| Dermatitis herpetiformis* | 0.104/0.057 | 0.046/0.044 | 0.055/0.046 | 0.101 | 0.300 | 0.263 |
| Localized scleroderma* | -0.026/0.049 | -0.004/0.044 | -0.024/0.046 | 0.648 | 0.925 | 0.642 |
| Pemphigoid* | 0.079/0.107 | 0.004/0.062 | -0.045/0.085 | 0.482 | 0.951 | 0.611 |
| Psoriasis | -0.105/0.187 | -0.053/0.118 | -0.061/0.123 | 0.588 | 0.656 | 0.631 |
| **7. Urologic disease** |  |  |  |  |  |  |
| IgA nephropathy* | 0.922/0.451 | 0.06/0.125 | 0.092/0.133 | 0.178 | 0.633 | 0.538 |

Abbreviations: Mendelian randomization (MR); inverse-variance weighted (IVW); Beta (b); standard error (se);

^a^ Genome-wide significance of the selected SNPs associated with the factors is less than 5×10-8, factors with * is less than 5×10-8.

* P<0.05

** P<0.01
