# Supplementary figures and images for: Genetic associations between autoimmune diseases and the risks of severe sepsis and 28-day mortality: a two-sample Mendelian randomization study
Source: Front Med (Lausanne). 2024 Jan 26;11:1331950. doi: 10.3389/fmed.2024.1331950 (PMC10853392; doi:10.3389/fmed.2024.1331950)

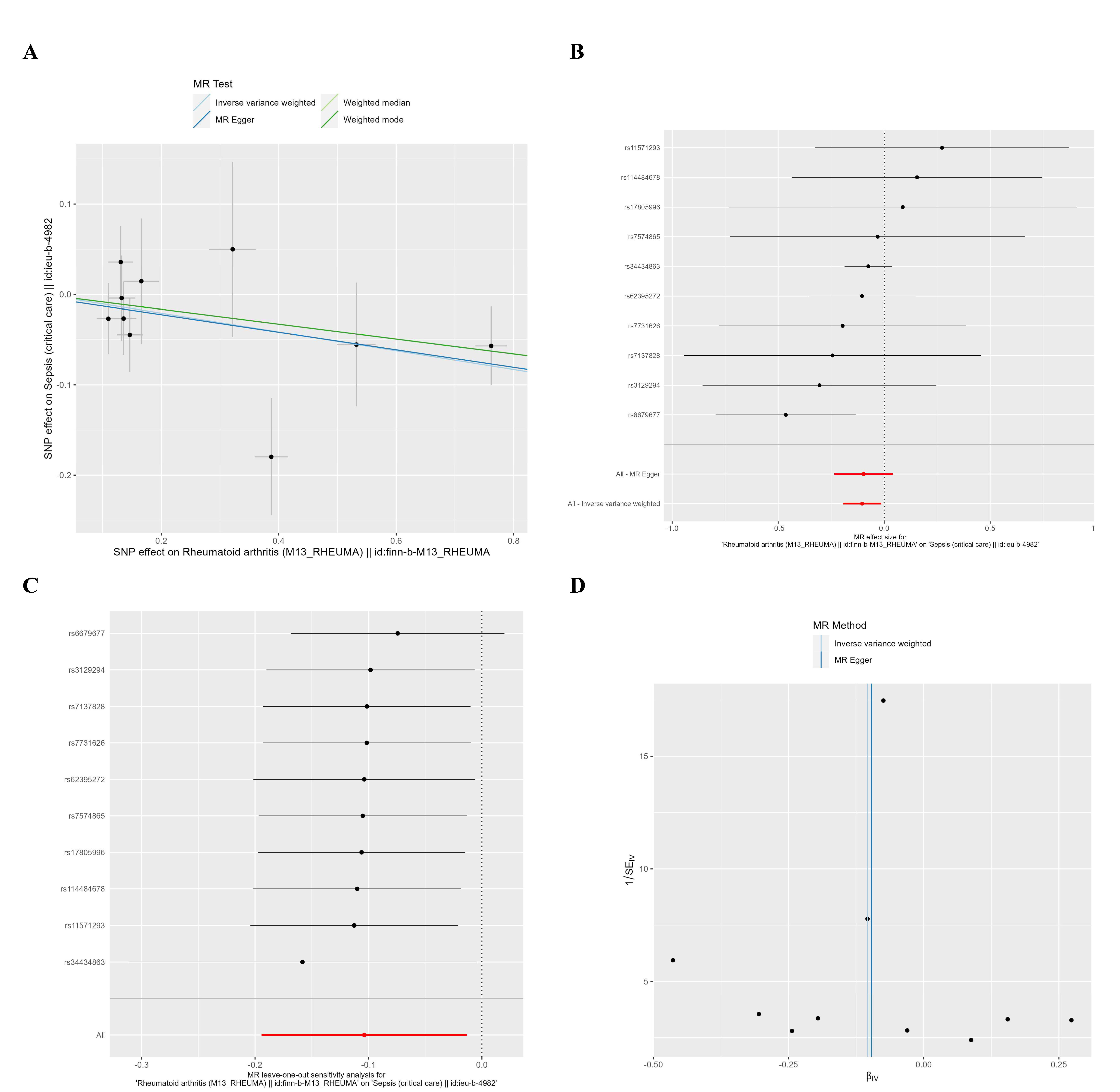

Supplement: Supplementary file 1 [file Data_Sheet_1.zip › Supplementary Figure 1.PNG]

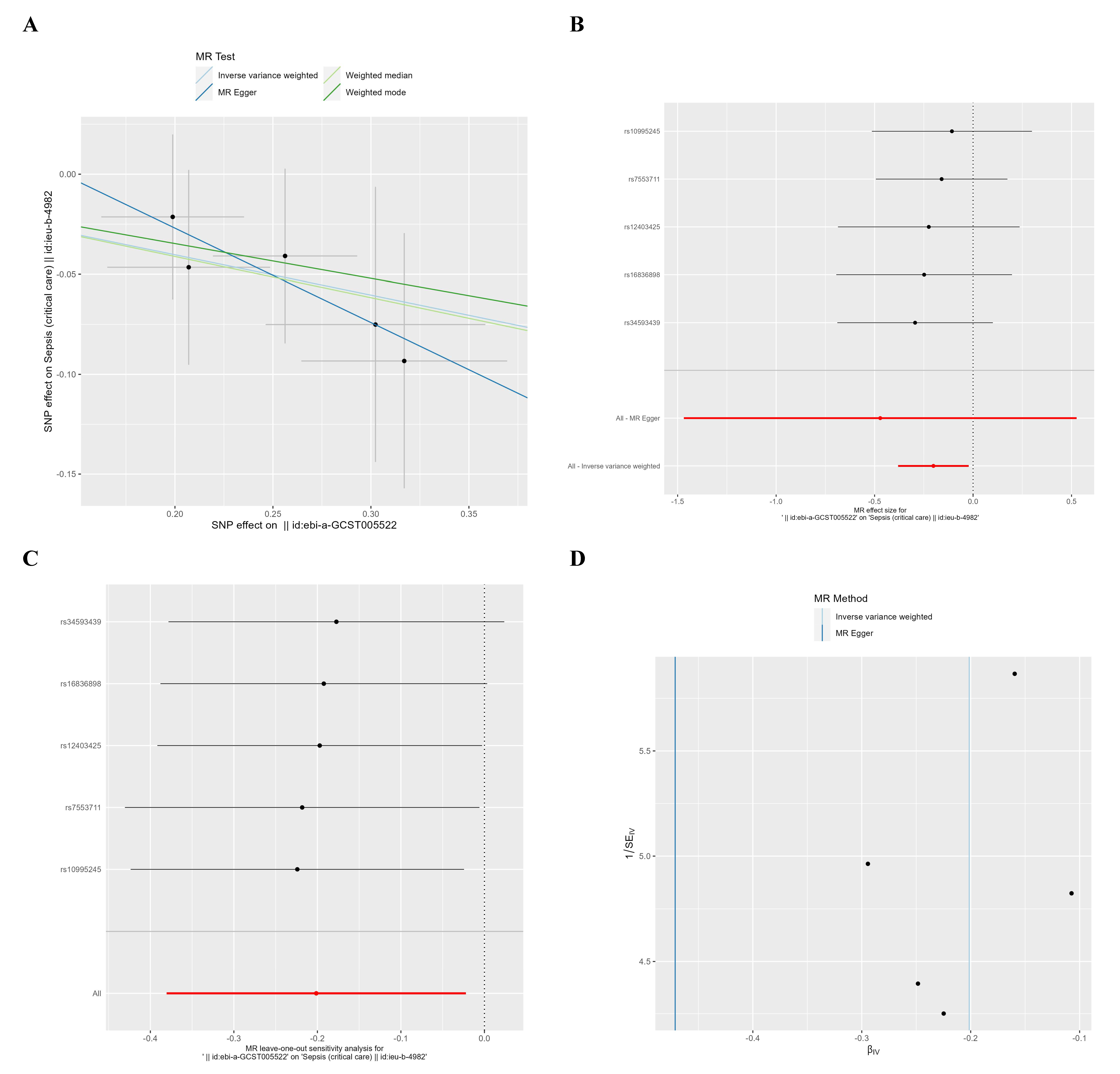

Supplement: Supplementary file 1 [file Data_Sheet_1.zip › Supplementary Figure 2.PNG]

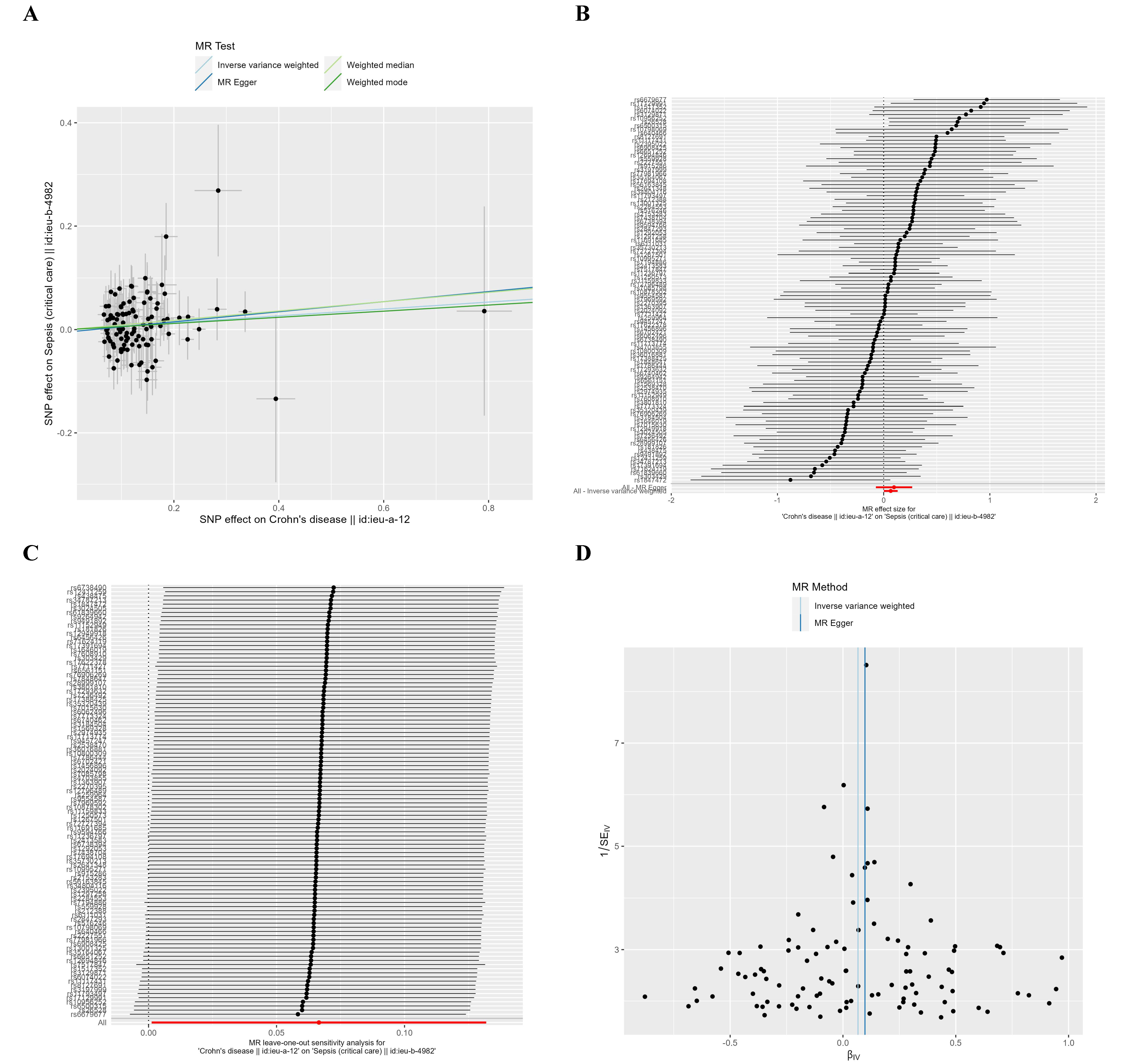

Supplement: Supplementary file 1 [file Data_Sheet_1.zip › Supplementary Figure 3.PNG]

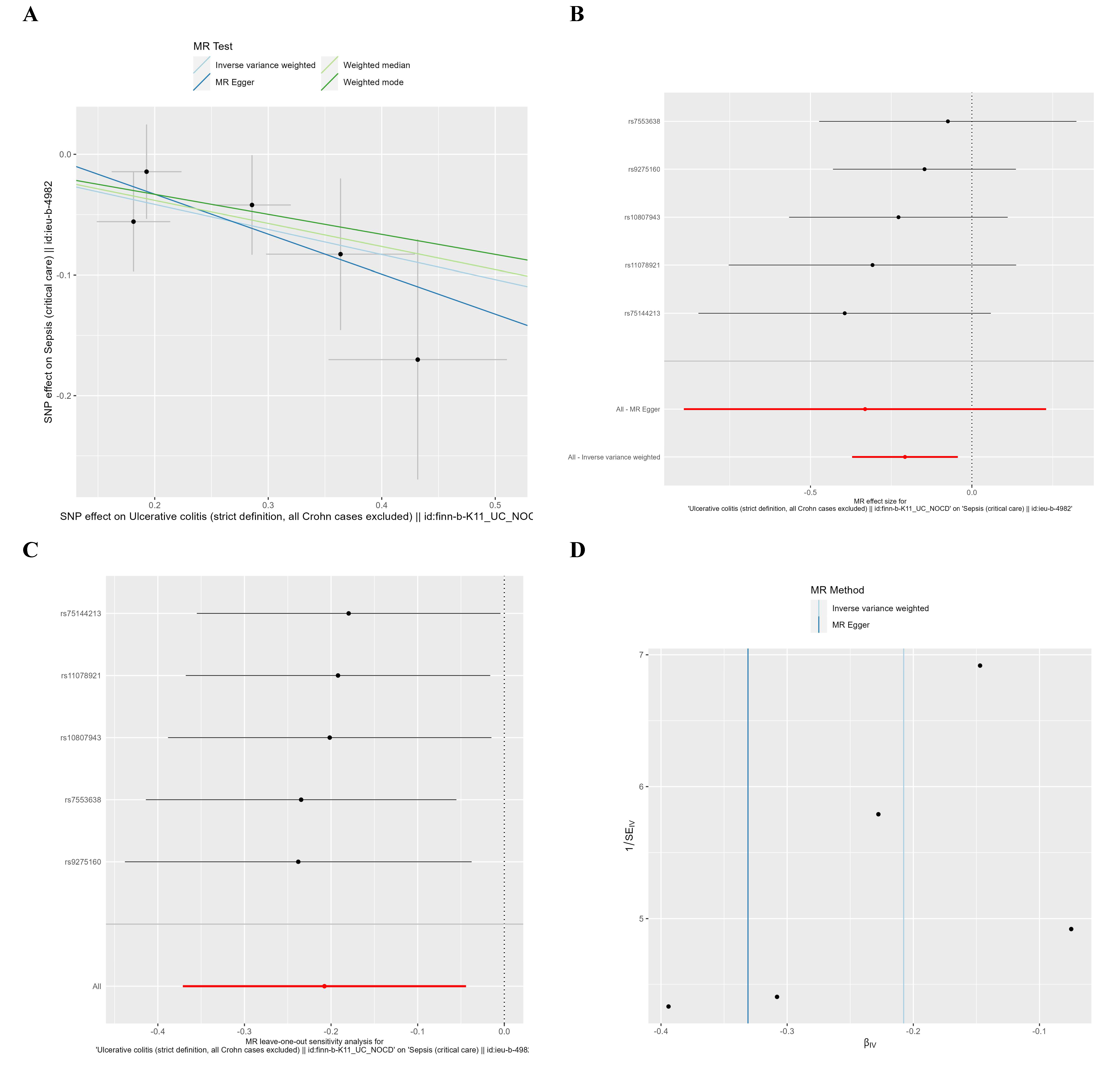

Supplement: Supplementary file 1 [file Data_Sheet_1.zip › Supplementary Figure 4.PNG]

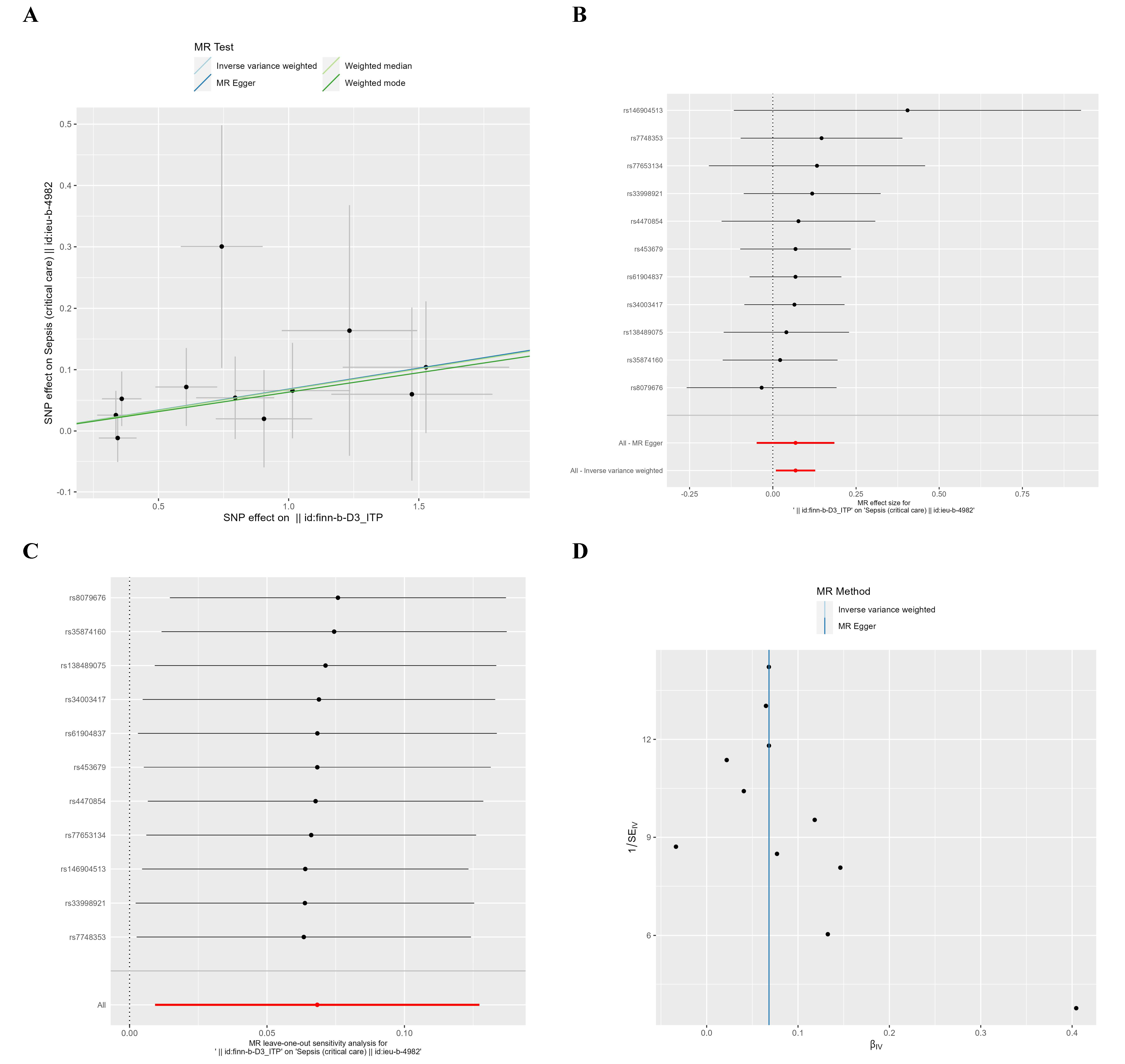

Supplement: Supplementary file 1 [file Data_Sheet_1.zip › Supplementary Figure 5.PNG]

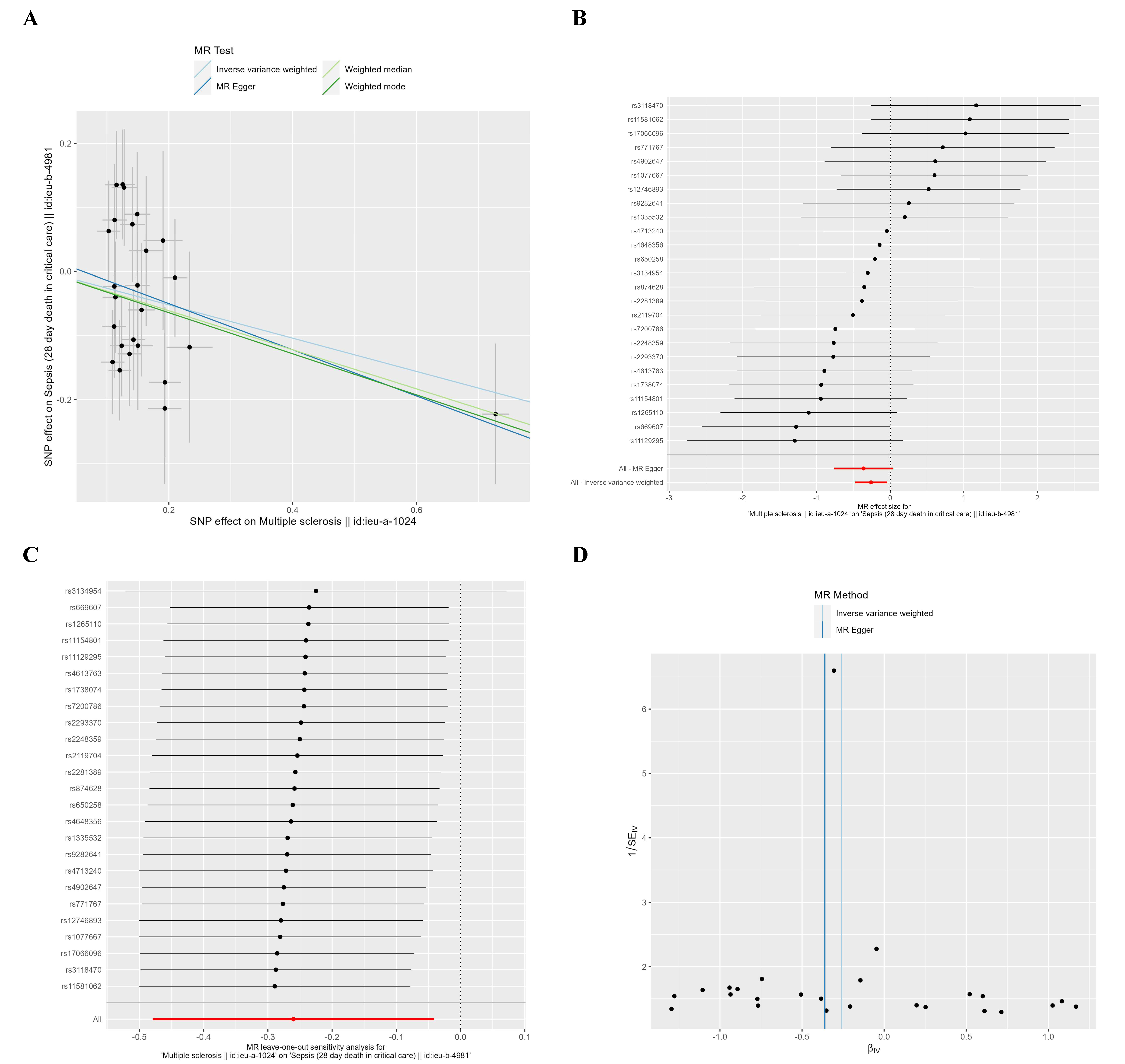

Supplement: Supplementary file 1 [file Data_Sheet_1.zip › Supplementary Figure 6.PNG]

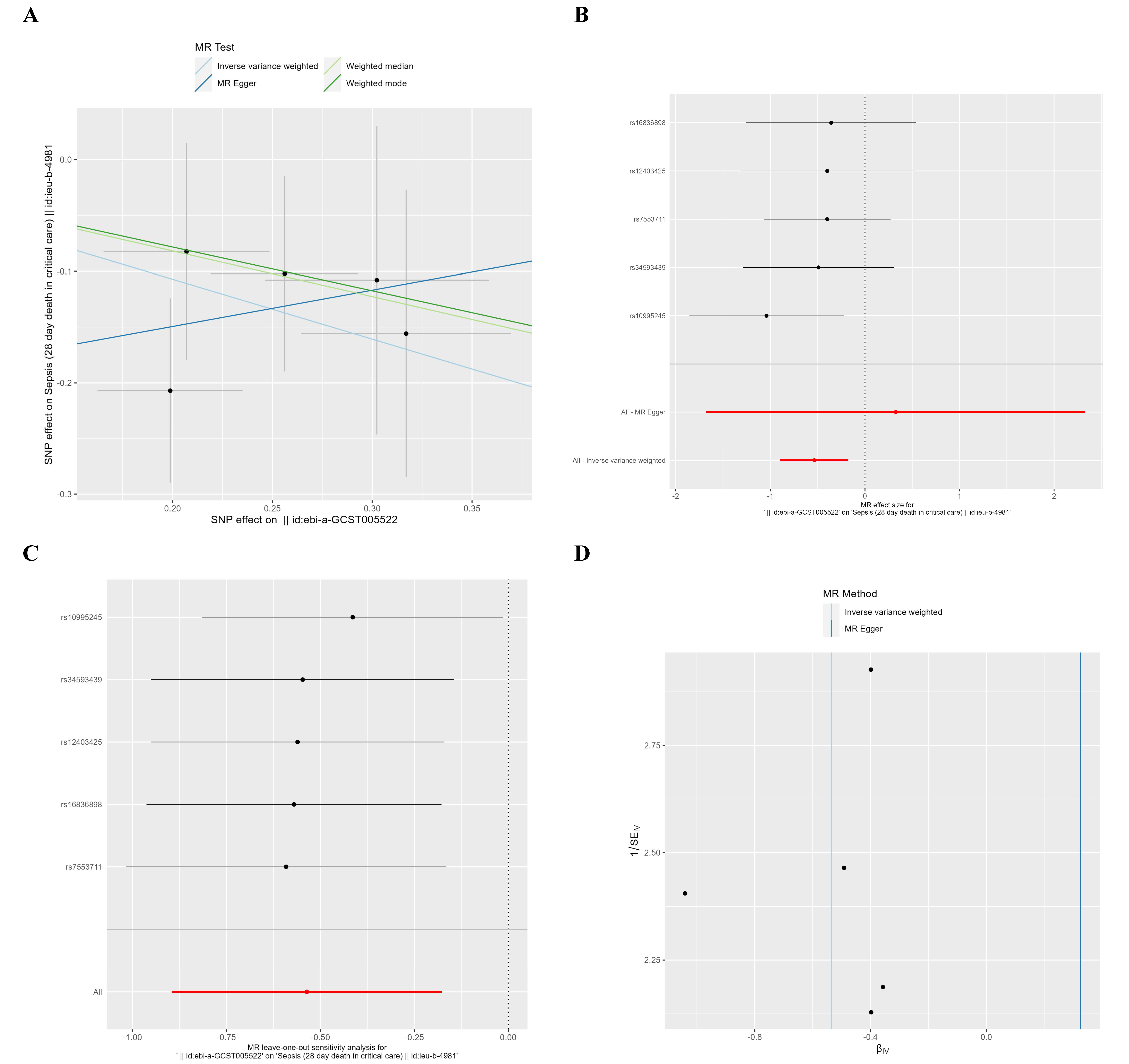

Supplement: Supplementary file 1 [file Data_Sheet_1.zip › Supplementary Figure 7.PNG]

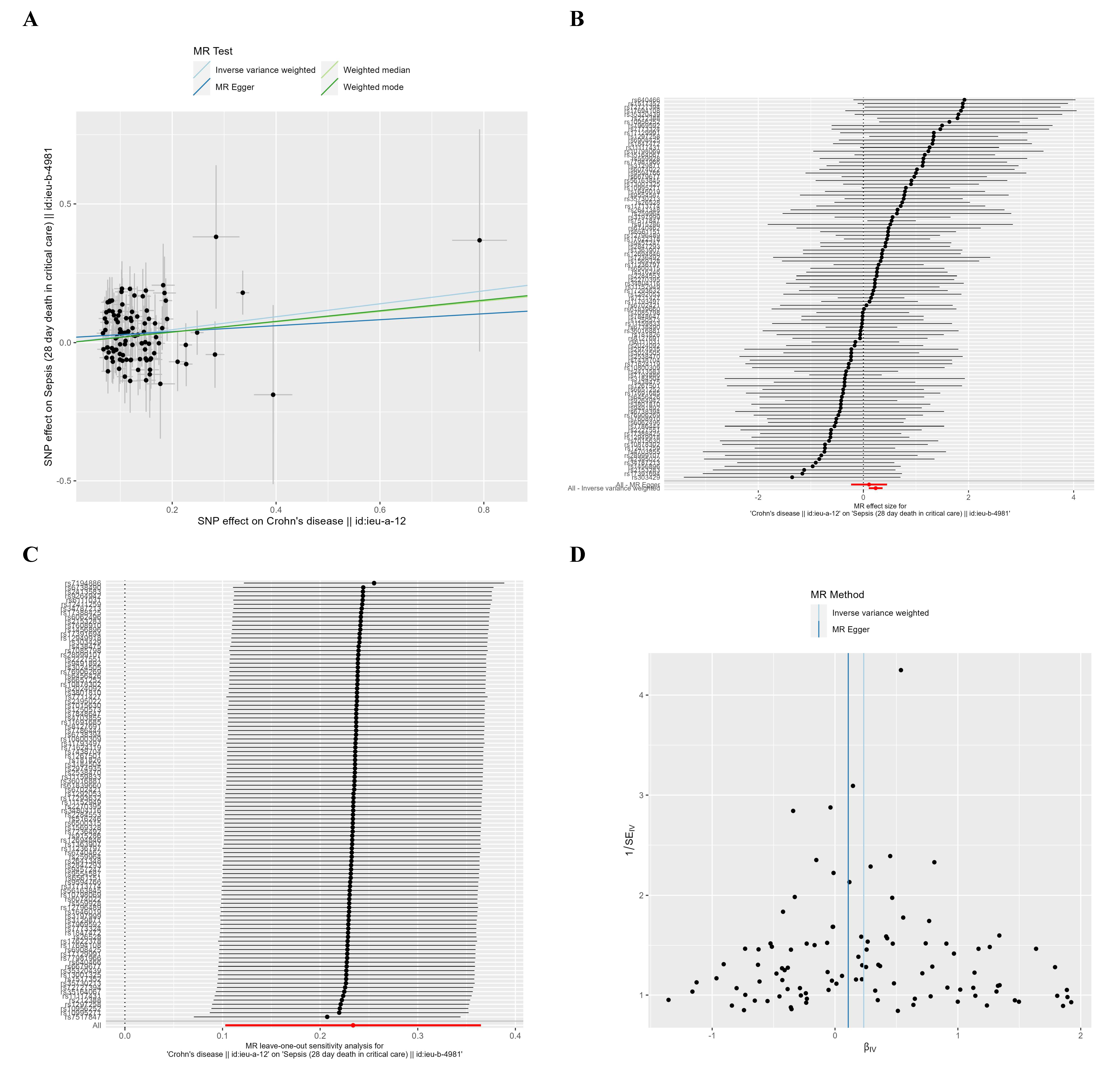

Supplement: Supplementary file 1 [file Data_Sheet_1.zip › Supplementary Figure 8.PNG]

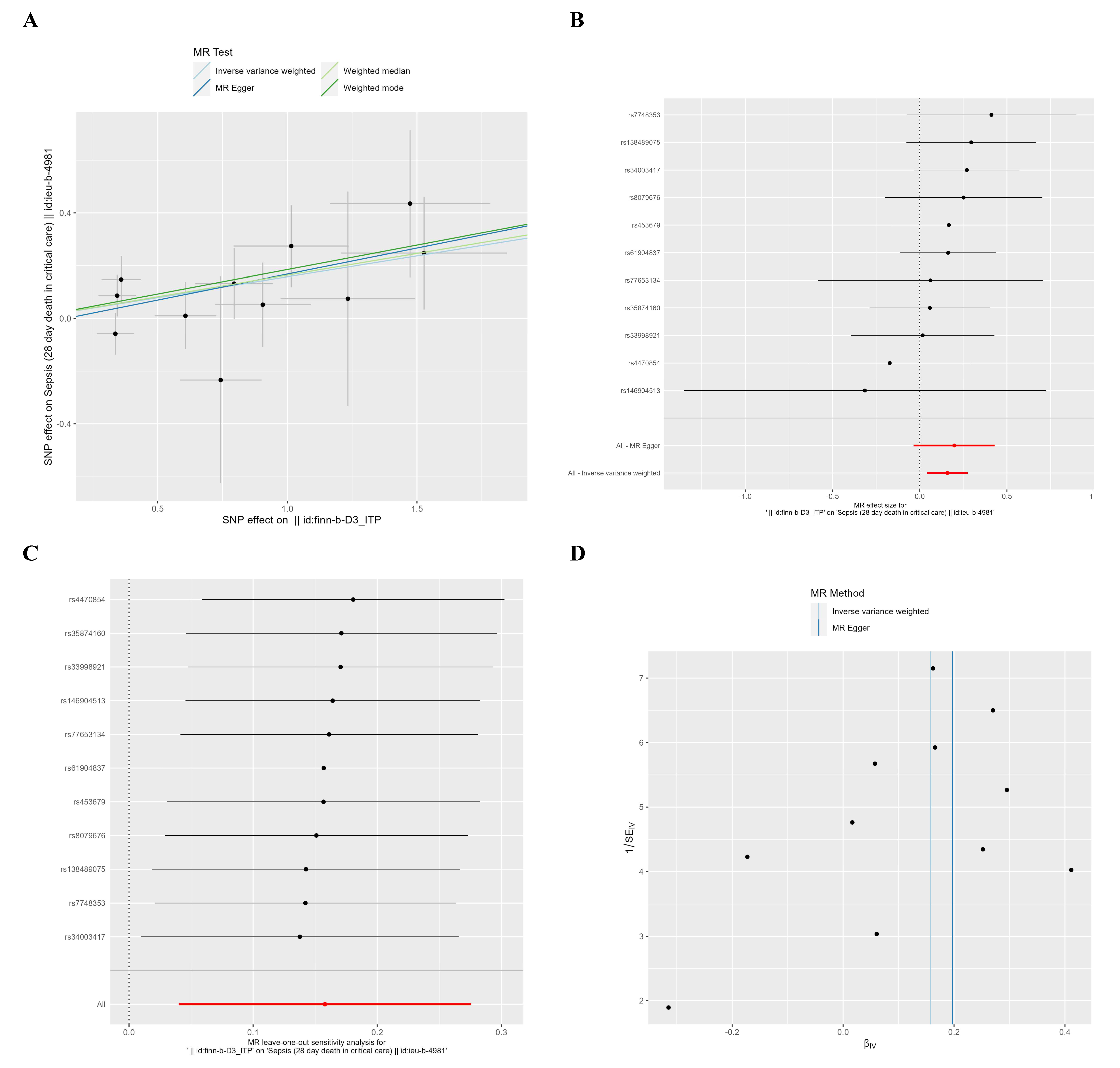

Supplement: Supplementary file 1 [file Data_Sheet_1.zip › Supplementary Figure 9.PNG]
